# Construction and validation of a nomogram for non small cell lung cancer patients with liver metastases based on a population analysis

**DOI:** 10.1038/s41598-022-07978-8

**Published:** 2022-03-07

**Authors:** Ruhan Zhao, Yunnan Dai, Xinyang Li, Cuimin Zhu

**Affiliations:** Department of Oncology, Hospital of Chengde Medical University, No.36 Nanyingzi St, Chengde, Hebei Province China

**Keywords:** Lung cancer, Cancer

## Abstract

Lung cancer is one of the most common malignancies in the United States, and the common metastatic sites in advanced non-small cell lung cancer (NSCLC) are bone, brain, adrenal gland, and liver, respectively, among which patients with liver metastases have the worst prognosis. We retrospectively analyzed 1963 patients diagnosed with NSCLC combined with liver metastases between 2010 and 2015. Independent prognostic factors for patients with liver metastases from NSCLC were identified by univariate and multivariate Cox regression analysis. Based on this, we developed a nomogram model via R software and evaluated the performance and clinical utility of the model by calibration curve, receiver operating characteristic curves, and decision curve analysis (DCA). The independent prognostic factors for NSCLC patients with liver metastases included age, race, gender, grade, T stage, N stage, brain metastases, bone metastases, surgery, chemotherapy, and tumor size. The area under the curve predicting OS at 6, 9, and 12 months was 0.793, 0.787, and 0.784 in the training cohort, and 0.767, 0.771, and 0.773 in the validation cohort, respectively. Calibration curves of the nomogram showed high agreement between the outcomes predicted by the nomogram and the actual observed outcomes, and the DCA further demonstrated the value of the clinical application of the nomogram. By analyzing the Surveillance, Epidemiology, and End Results database, we established and verified a prognostic nomogram for NSCLC patients with liver metastases, to personalize the prognosis of patients. At the same time, the prognostic nomogram has a satisfactory accuracy and the results are a guide for the development of patient treatment plans.

## Introduction

Lung cancer is currently the most common and deadliest malignancy in the world, and the number of new diagnoses and deaths of lung cancer patients in the United States is expected to be 235,760 and 131,880, respectively, in 2021, according to cancer data published in the United States^1^. Lung cancer can be divided into two categories: non-small cell lung cancer (NSCLC) and small cell lung cancer, and NSCLC account for approximately 85% of lung cancer cases, due to clinical manifestations and signs are not obvious, and about 40%-55% of these patients are advanced at the time of diagnosis^2^. The common sites of metastasis in advanced NSCLC are bone, brain, adrenal gland, and liver^3^. For advanced patients presenting with metastases, their median survival is 8–12 months, while in patients with metastases at common sites, the median Overall survival(OS) is only 3 months once liver metastases are diagnosed^4,5^. It has also been demonstrated that liver metastasis is one of the independent poor prognostic factors for patients with NSCLC, and therefore patients with liver metastases from NSCLC have the worst prognosis^6^. TNM staging is now widely accepted as a tool to predict patient prognosis^7^. However, the patient's age, gender, marital status, pathological features, distant metastatic sites and numbers, and treatment methods all have an impact on their prognosis^8^.Therefore, it is still difficult to accurately predict the prognosis of metastatic lung cancer using TNM staging.

Compared with brain metastases and bone metastases, the prognosis of patients with liver metastases from non-small cell lung cancer has been less studied, and there is no effective model to predict the prognosis of NSCLC patients with liver metastases. The nomogram is a simple statistical tool that can meet our needs for a comprehensive clinical model. Nomograms enable rapid calculations as well as higher accuracy and easier to understand prognosis compared to traditional TNM staging to assist physicians in clinical decision making^9^. Therefore, through this study, we aimed to integrate the clinical characteristics of patients in the Surveillance, Epidemiology, and End Results database (SEER) database between 2010 and 2015 and quantify the impact of each risk to create a nomogram, which can make the results of the prediction model more readable through visual graphs and can more accurately determine the OS of NSCLC patients with liver metastases, helping clinicians to be able to better develop treatment strategies.

## Methods

### Patient selection

We screened patients diagnosed with NSCLC combined with liver metastases between 2010 and 2015 in the SEER database. Because patient information in the SEER database is publicly available and free of charge, institutional review board approval was not required for this study. Inclusion criteria were: (1) patients aged ≥ 18 years, (2) patients whose only primary site tumor was pathologically diagnosed as NSCLC, (3) those with complete information, including race, primary site, grade, marital status, surgery, chemotherapy, radiation therapy, and insurance status. We finally screened 1963 NSCLC patients with liver metastases for inclusion in this study.

### Variable definitions

We extracted factors from the patient data that might be associated with prognosis, including age, gender, race, laterality, primary site, histological type, grade, T stage, N stage, surgery, radiotherapy, chemotherapy, bone metastases, brain metastases, lung metastases, marital status, and insurance status. Age was changed from a continuous variable to a categorical variable by X-tile software and divided into < 55, 55–66, and > 66. Similarly, the tumor size is divided into < 42, 42–71 and > 71. The tumor site was divided into the main bronchus, upper lobe, middle lobe, lower lobe, and overlapping sites based on anatomy. According to the 8th edition of the American Joint Committee on cancer guidelines, T was divided into T1, T2, T3, and T4, and similarly, N was divided into N1, N2, and N3. The primary endpoint of this study was overall survival (OS), defined as the time interval from the date of diagnosis to the date of patient death.

### Statistical analysis

We randomly divided patients into a training group (n = 1375) and a validation group (n = 588) in a ratio of 7:3. The variables associated with prognosis in NSCLC with liver metastases were identified by univariate Cox regression analysis of prognosis-related indicators. Subsequently, variables with P values < 0.05 in the univariate Cox regression analysis were subjected to multivariate Cox regression analysis to obtain independent prognostic factors for NSCLC with liver metastases, and multiple predictors were integrated to assign different scores using the degree of contribution of different factors to OS. Finally, a nomogram model for predicting OS in NSCLC patients with liver metastases was established by converting the scores to a functional relationship with OS using R software. The model performance is divided into two main aspects, discrimination and calibration, which we have validated in the training and validation groups, respectively. We used calibration curves to measure the agreement between predicted and actual outcomes. The discriminant of the model was measured by calculating the receiver operating characteristic curves (ROC) curves area under the curve (AUC), which took values in the range of 0.5–1.0. Finally, the clinical application value of the model was evaluated by decision curve analysis (DCA). The random grouping, nomogram, calibration curves, ROC, and DCA were composed by R language software (version 4.0.3).The cox proportional hazards regression analyses were performed by the R software “survival” and “MASS” packages. The Kaplan–Meier survival curve was produced using the “tidyverse”and “survminer” package.The nomogram and calibration plots were produced using the “rms” “foreign”and “survival” package. The DCA was performed using the function “stdca.R”.The ROC was performed using the “survival”package. Bilateral P values < 0.05 were considered statistically significant.

## Results

### Baseline characteristics of NSCLC patients with liver metastases

A total of 199,564 patients with confirmed NSCLC were collected in the SEER database between 2010 and 2015, and 1963 eligible cases were finally obtained, of which 1375 were randomly assigned to the training group and 588 were randomly assigned to the validation group. Table 1 summarizes the demographic and clinical characteristics of the NSCLC patients with liver metastases. In the training group, 733 (53.3%) patients were > 66 years old, 1073 (78%) were white, 804 (58.4%) were male, and 1330 (96.7%) patients had insurance. For tumor characteristics, 54.6% of them were adenocarcinoma, 59.2% were upper lobe, 68.4% were histologic grade III, and 50.2% were N2. Among the modalities of follow-up treatment received, the majority (97%) of patients did not receive surgical treatment, 55.7% received chemotherapy, and 42.9% received radiation therapy. Patient details are shown in Table 1.

**Table 1:** Demographic and clinical characteristics of patients.

Variables	Training cohort		Validation cohort	
	N = 1375		N = 588	
	n	%	n	%
**Age**				
< 55	162	11.7	89	15.1
55–66	480	35.0	200	34.0
> 66	733	53.3	299	50.9
> 66	733			
**Race**				
Black	144	10.5	57	9.7
Other	158	11.5	59	10.0
White	1073	78.0	472	80.3
**Sex**				
Male	804	58.5	338	57.5
Female	571	41.5	250	42.5
**Primary site**				
Lower lobe	413	30.1	192	32.7
Main bronchus	63	4.6	26	4.4
Middle lobe	68	4.9	31	5.3
Overlapping lesion of lung	17	1.2	7	1.2
Upper lobe	814	59.2	332	56.4
**Laterality**				
Left	613	44.6	213	36.2
Right	762	55.4	375	63.8
**Histological types**				
Adenocarcinoma	752	54.7	322	54.8
Other	245	17.8	109	18.5
Squamous cell carcinoma	378	27.5	157	26.7
**Grade**				
I	65	4.7	26	4.4
II	335	24.4	144	24.5
III	941	68.4	401	68.2
IV	34	2.5	17	2.9
**T stage**				
T1	112	8.1	51	8.7
T2	388	28.2	156	26.5
T3	395	28.7	176	29.9
T4	480	35.0	205	34.9
**N stage**				
No	272	19.8	115	19.6
N1	122	8.9	42	7.1
N2	691	50.2	293	49.8
N3	290	21.1	138	23.5
**Surgery**				
No	41	3.0	568	96.6
Yes	1334	97.0	20	3.4
**Chemotherapy**				
No	608	44.2	247	42.0
Yes	767	55.8	341	58.0
**Radiotherapy**				
No	785	57.1	335	57.0
Yes	590	42.9	253	43.0
**Bone metastasis**				
No	667	48.5	278	47.3
Yes	708	51.5	310	52.7
**Brain metastasis**				
No	1004	73.0	442	75.2
Yes	371	27.0	146	24.8
**Lung metastasis**				
No	881	64.1	384	65.3
Yes	494	35.9	204	34.7
**Tumor size**				
< 42	521	37.9	227	38.6
42–71	555	40.4	219	37.2
> 71	299	21.7	142	24.2
**Insurance status**				
No	45	3.3	12	2.0
Yes	1330	96.7	576	98.0
**Marital**				
No	608	44.2	269	45.7
Yes	767	55.8	319	54.3

### Development and validation of a prognostic nomogram for NSCLC patients with liver metastases

**Figure 1:**
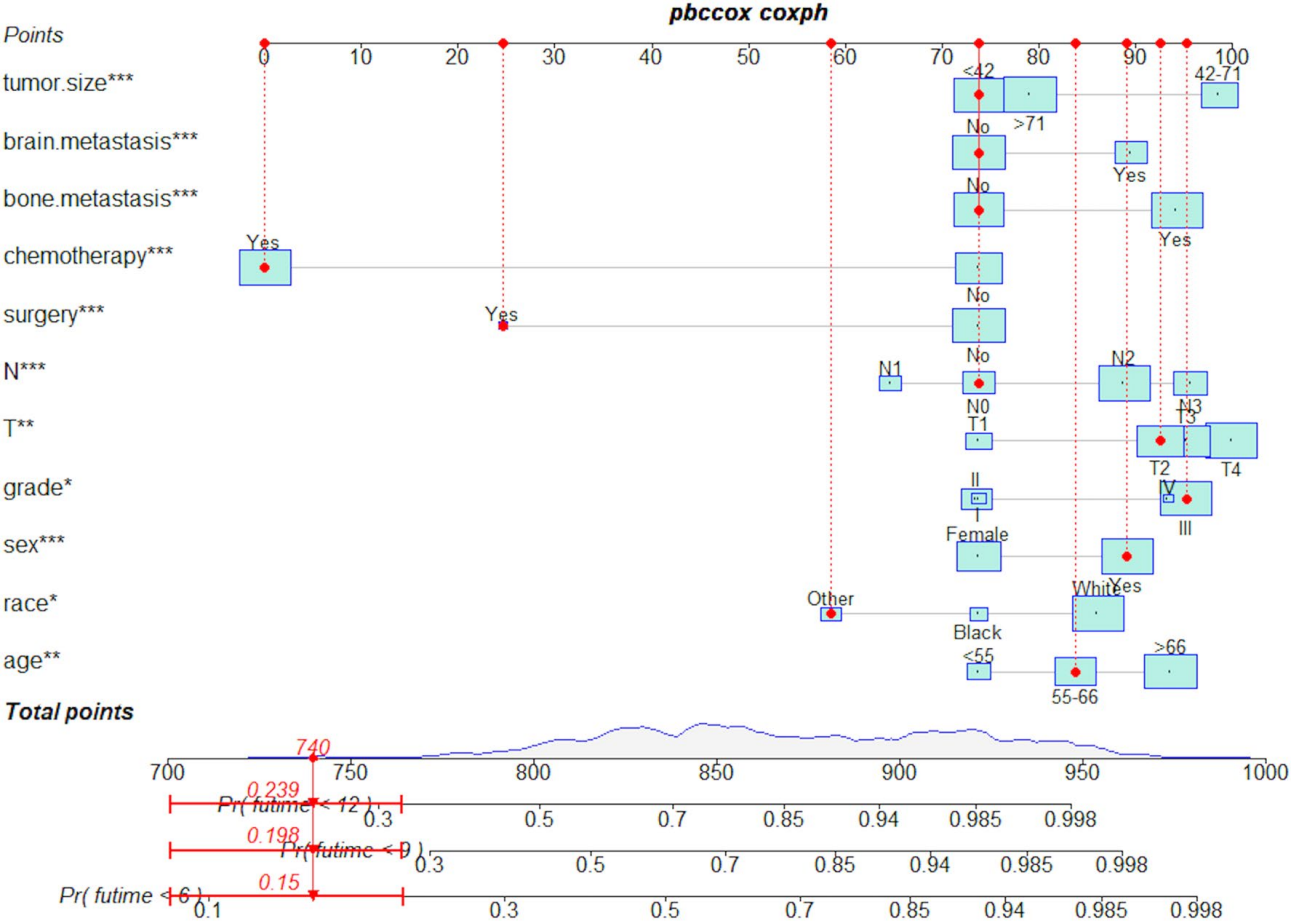
A prognostic nomogram for NSCLC patients with liver metastases.

**Figure 2:**
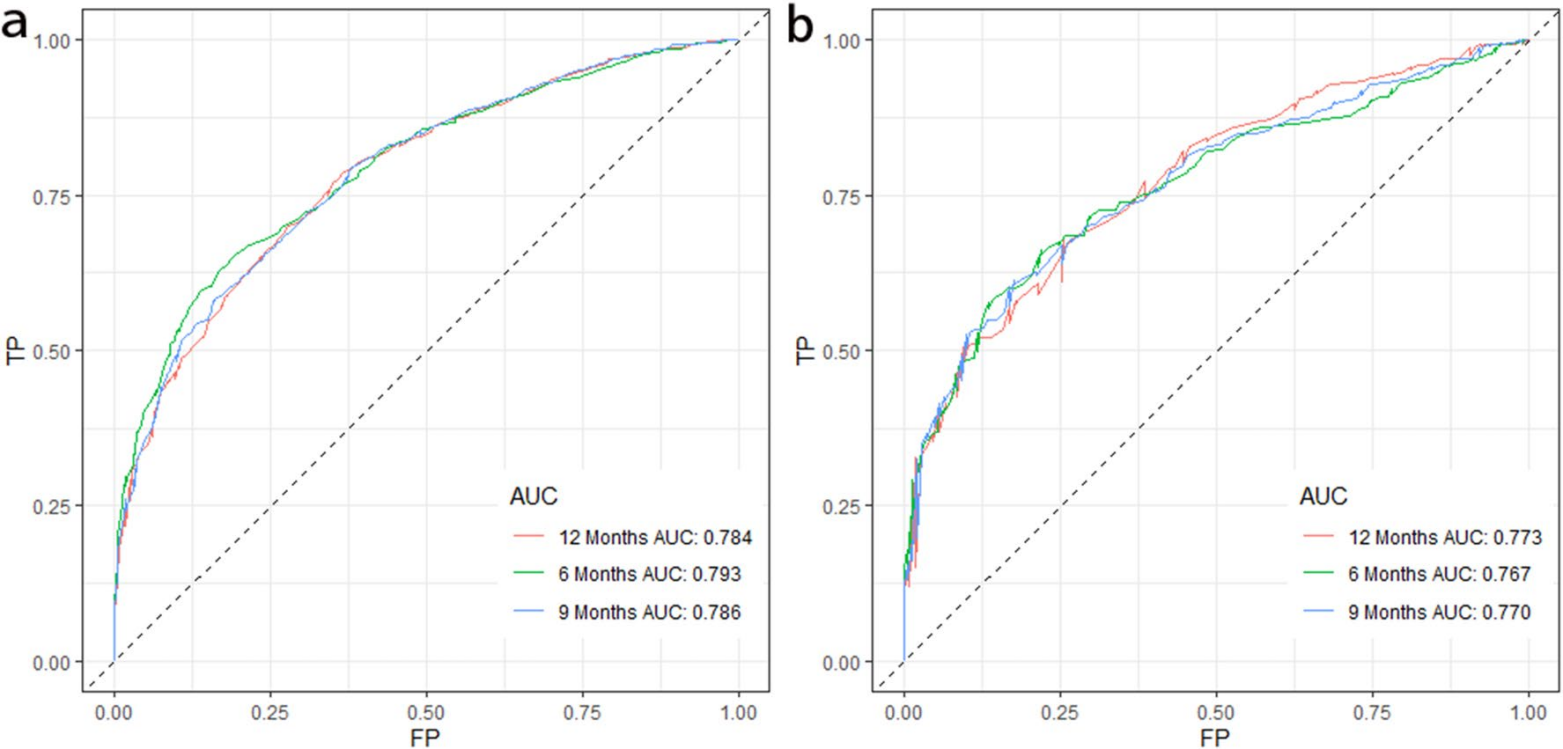
ROC curves for NSCLC patients with liver metastases. (**a**) ROC curves of 6-, 9-, and 12-months in the training group, (**b**) ROC curves of 6-, 9-, and 12-months in the validation group. ROC: Receiver operating characteristic.

**Figure 3:**
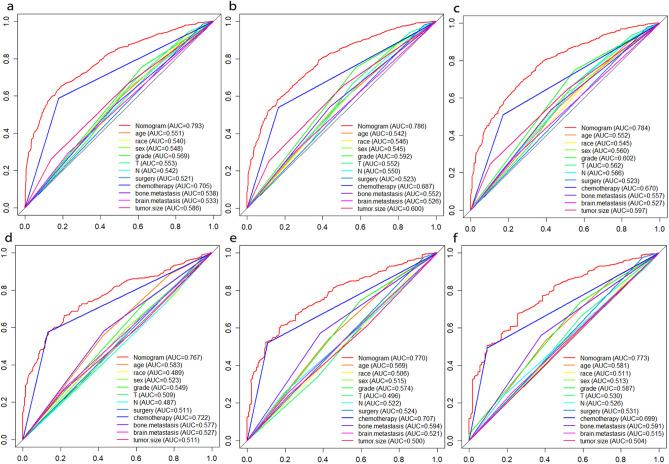
The ROC curves of nomogram and all independent predictors at 6- (**a**), 9- (**b**), and 12-months (**c**) in the training group and at 6- (**d**), 9- (**e**), and 12-months (**f**) in the validation group.

**Figure 4:**
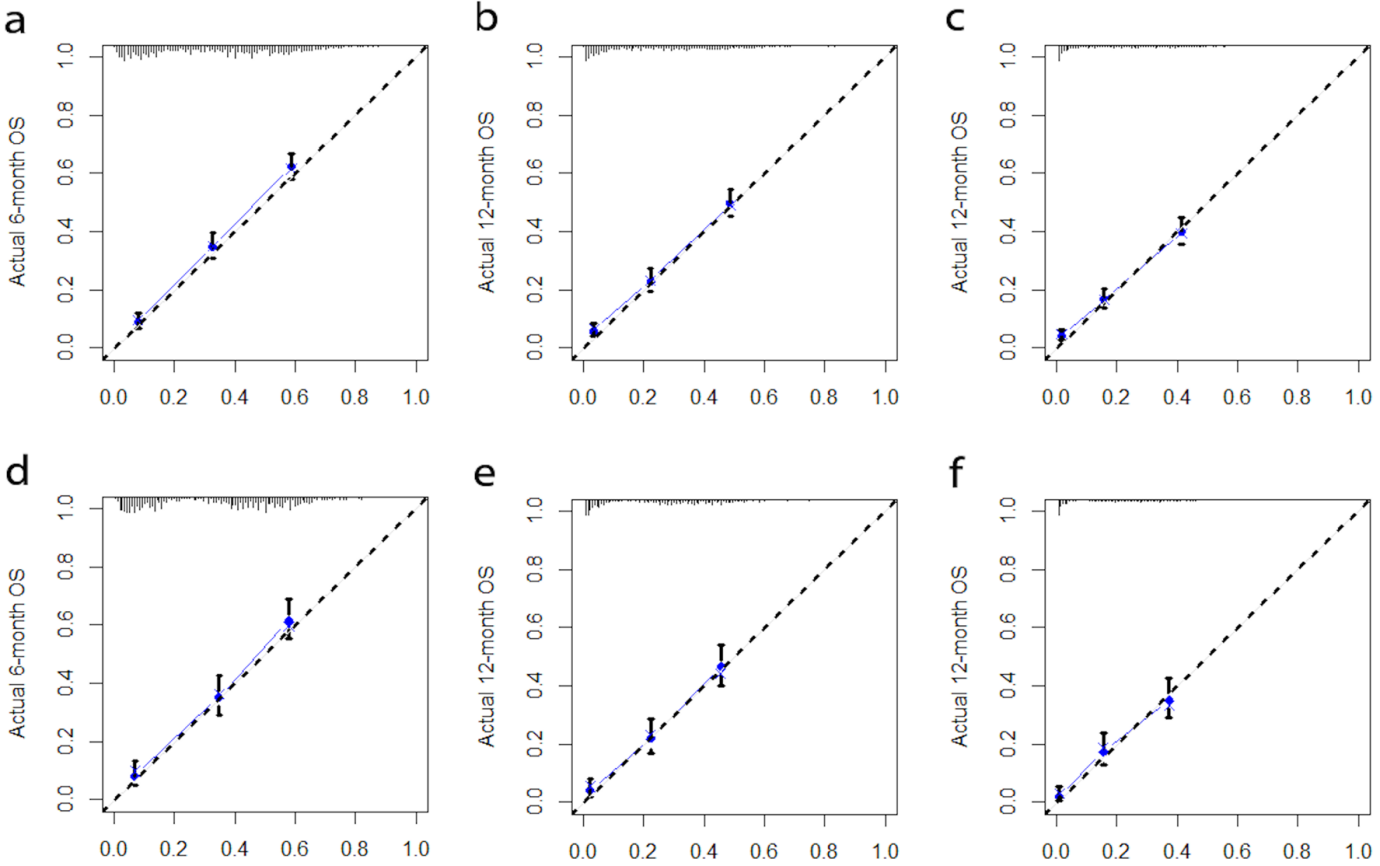
The calibration curves of the nomogram for the 6-, 9-, and 12-months OS prediction of the training group (**a**–**c**) and validation group (**d**–**f**). The x axis represents the nomogram- predicted survival rates, whereas the y axis represents the actual survival rates.

**Figure 5:**
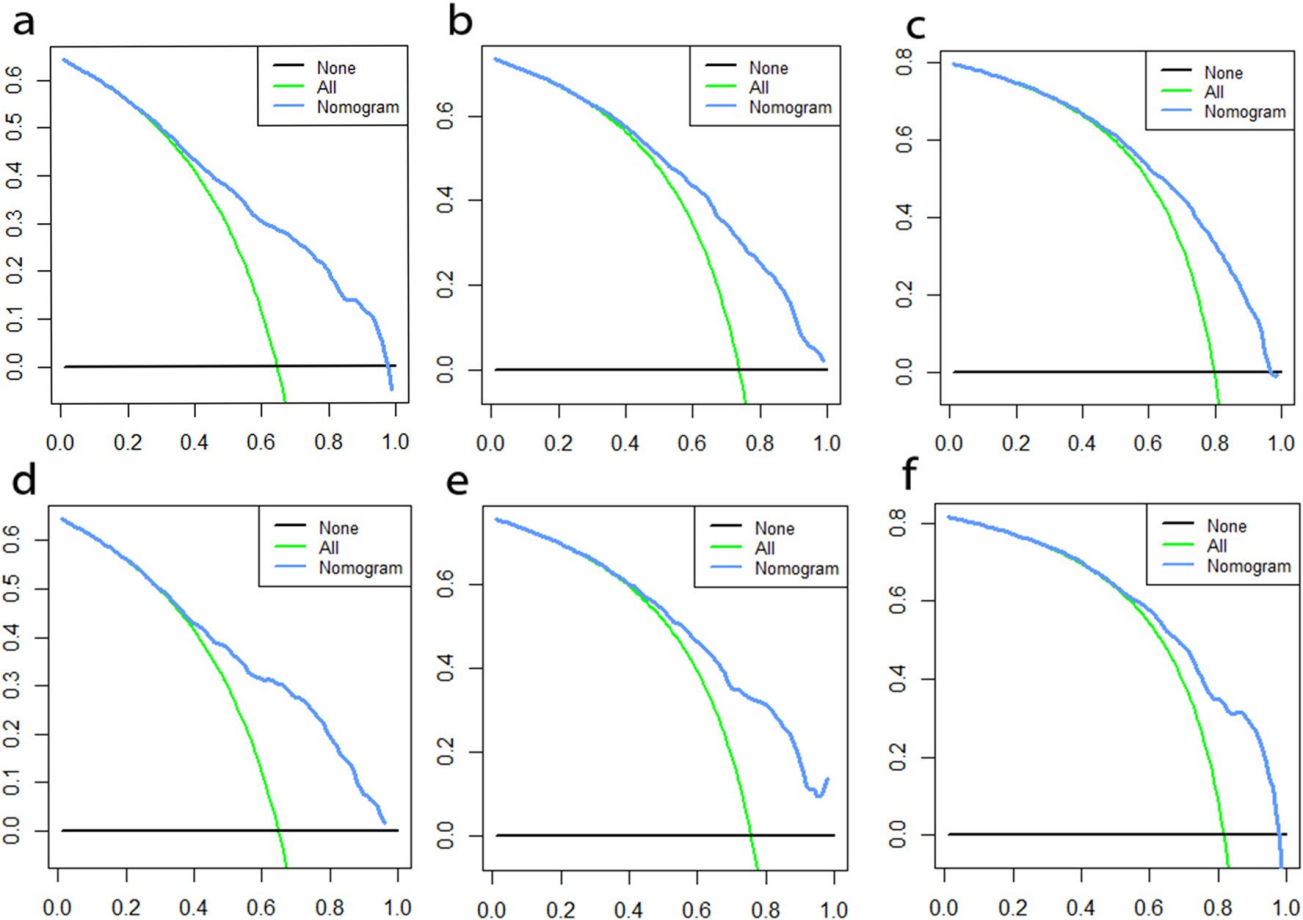
DCA of the nomogram for the survival prediction of NSCLC patients with liver metastases.6-months (**a**), 9-months (**b**), 12-months (**c**) survival benefit in the training group. 6-months (**d**), 9-months (**e**), 12-months (**f**) survival benefit in the validation group. The green curve represents the assumption that all patients will expire, while the blue curve indicates the net benefit of the nomogram model by summing the true positives and subtracting the false positives.

**Figure 6:**
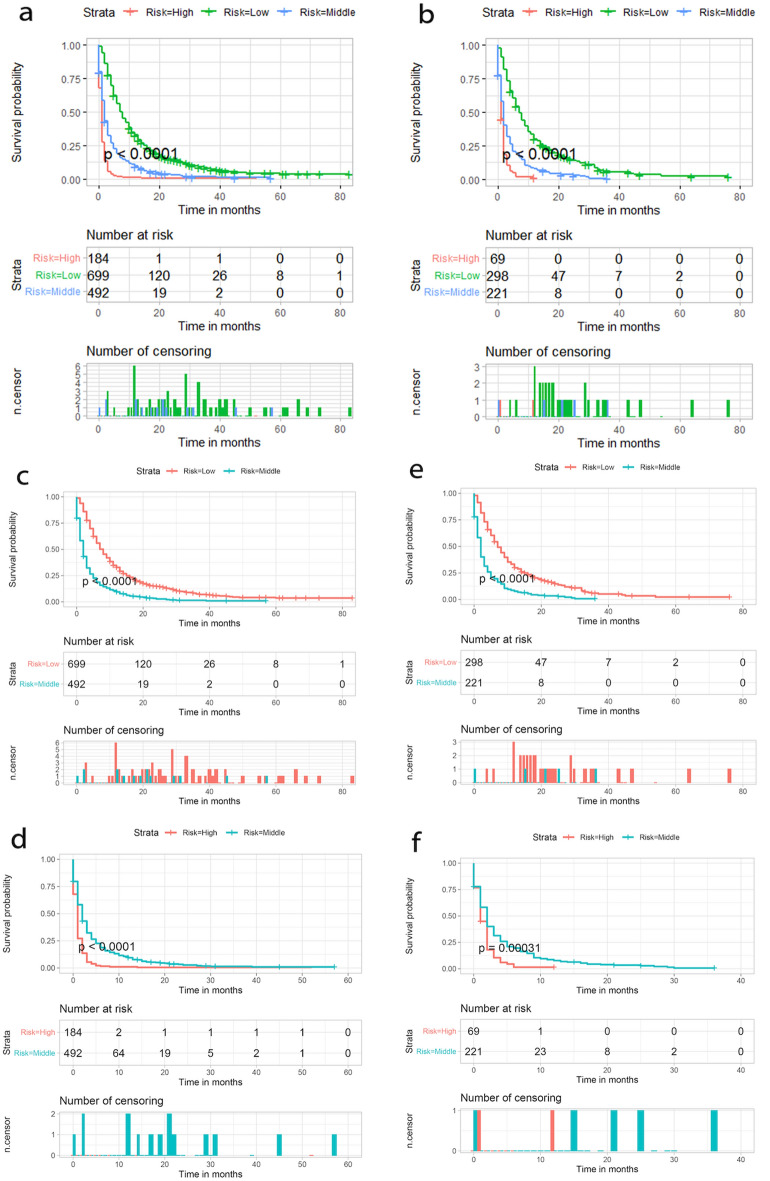
Kaplan–Meier survival analysis of the signature for both the training group and the validation group. Patients with a higher risk score demonstrated a worse OS than those with a low risk score in the training group (**a c d**) and validation group (**b**, **e**, **f**), which suggests the strong predictive ability for NSCLC patients with liver metastases survival outcome.

After univariate Cox regression analysis, a total of 14 factors were significantly associated with the prognosis of NSCLC patients with liver metastases (P < 0.05), including age, race, gender, histological type, grade, T stage, N stage, brain metastases, bone metastases, lung metastases, surgery, chemotherapy, insurance status, and tumor size (Table 2). These factors were then subjected to multivariate Cox regression analysis, and finally, 11 factors were identified as independent prognostic factors for NSCLC with liver metastases including age, race, gender, grade, T stage, N stage, brain metastases, bone metastases, surgery, chemotherapy, and tumor size. We established a prognostic nomogram for NSCLC patients with liver metastases based on the independent prognostic factors selected in the multivariate Cox regression analysis (Fig. 1), from which it is clear that the T stage has the greatest impact on the prognosis of NSCLC patients with liver metastases, followed by tumor size. The area under the curve of the clinical prognostic model predicting overall survival at 6, 9, and 12 months was 0.793, 0.787, and 0.784 in the training group and 0.767, 0.771, and 0.773 in the validation group, respectively (Fig. 2). In addition, we further compared the difference of AUC value between nomogram and all independent prognostic factors and the results showed that the AUC value of the nomogram was higher than the AUC of all independent factors at 6, 9, and 12 months, both in the training group and the validation group (Fig. 3). The calibration curves are shown in Fig. 4. The curves are all close to 45 degrees, indicating that the prognostic nomogram has a good calibration performance. Also, the DCA showed that the prognostic nomogram has strong clinical utility (Fig. 5). We calculated the total score for all patients and then used X-tile software to cutoff. The results showed that the best cut-off values for OS were 105 and 167. Based on the cut-off values, patients were divided into the high-risk group (> 167), medium-risk group (106–166), and low-risk group (< 106). By depicting the Kaplan–Meier survival curve, we can find that as the risk increases, the prognosis of the patients will become worse. (Fig. 6).

**Table 2:** Univariate and multivariate Cox regression analysis in NSCLC patients with liver metastases.

	Univariate Cox analysis				Multivariate Cox analysis			
	HR	95%CI		*P*	HR	95%CI		*P*
**Age**								
< 55	1				1			
55–66	1.212	1.006	1.460	0.043	1.151	0.954	1.390	0.143
> 66	1.383	1.157	1.653	< 0.001	1.316	1.097	1.579	0.003
**Race**								
Black	1				1			
Other	0.724	0.572	0.917	0.007	0.803	0.632	1.022	0.074
White	1.030	0.862	1.231	0.746	1.178	0.981	1.414	0.079
**Sex**								
Female	1				1			
Male	1.289	1.153	1.441	< 0.001	1.235	1.103	1.384	< 0.001
**Primary site**								
Lower lobe	1							
Main bronchus	1.086	0.826	1.428	0.555				
Middle lobe	0.873	0.664	1.148	0.330				
Overlapping lesion of lung	1.624	0.984	2.680	0.058				
Upper lobe	1.023	0.905	1.156	0.718				
**Laterality**								
Left	1							
Right	0.954	0.855	1.065	0.400				
**Histological types**								
Adenocarcinoma	1							
Other	1.290	1.111	1.498	0.001				
Squamous cell carcinoma	1.272	1.119	1.445	< 0.001				
**Grade**								
I	1				1			
II	1.160	0.871	1.544	0.310	1.003	0.747	1.347	0.985
III	1.673	1.273	2.198	< 0.001	1.362	1.026	1.807	0.032
IV	1.708	1.104	2.643	0.016	1.333	0.853	2.084	0.207
**T stage**								
T1	1				1			
T2	1.460	1.168	1.825	0.001	1.317	1.035	1.676	0.025
T3	1.735	1.389	2.167	< 0.001	1.367	1.067	1.752	0.014
T4	1.725	1.386	2.148	< 0.001	1.449	1.137	1.847	0.003
**N stage**								
No	1				1			
N1	0.933	0.744	1.168	0.544	0.877	0.696	1.103	0.262
N2	1.166	1.007	1.349	0.040	1.221	1.046	1.424	0.011
N3	1.262	1.064	1.496	0.007	1.336	1.115	1.602	0.002
**Surgery**								
No	1				1			
Yes	0.399	0.275	0.580	< 0.001	0.484	0.330	0.711	< 0.001
**Chemotherapy**								
No	1				1			
Yes	0.407	0.364	0.456	< 0.001	0.365	0.324	0.411	< 0.001
**Radiotherapy**								
No	1							
Yes	0.990	0.887	1.105	0.857				
**Bone metastasis**								
No	1				1			
Yes	1.226	1.098	1.368	< 0.001	1.323	1.179	1.484	< 0.001
**Brain metastasis**								
No	1				1			
Yes	1.156	1.024	1.307	0.020	1.232	1.086	1.398	0.001
**Lung metastasis**								
No	1							
Yes	1.198	1.069	1.341	0.002				
**Tumor size**								
< 42	1				1			
42–71	1.207	1.065	1.368	0.003	1.078	0.944	1.232	0.267
> 71	1.715	1.480	1.987	< 0.001	1.410	1.195	1.664	< 0.001
**Insurance status**								
No	1							
Yes	0.693	0.511	0.940	0.019				
**Marital**								
No	1							
Yes	0.901	0.807	1.006	0.064

## Discussion

The incidence of liver metastases in lung cancer ranges 2.9–4.1% and is 20–30% in patients with NSCLC, which makes the prognosis worse^10^. The risk of death in patients with NSCLC in the presence of liver metastases is 1.53–2.41 times higher than that of distant metastases from other sites^8,11^. However, there is no relevant model that can personalize the prognosis of patients with liver metastases from NSCLC. Therefore, we created a prognostic nomogram for NSCLC patients with liver metastases by analyzing the basic information of patients in the SEER database and achieved a more precise to determine the prognosis of patients by corresponding each variable to the nomogram graph. The study confirmed that race, age, gender, grade, T stage, N stage, tumor size, presence of distant metastases from remaining sites (e.g., brain, bone, etc.), surgery, and chemotherapy were all associated with prognosis.

The results of this study showed that females had a better prognosis compared to males in NSCLC patients with liver metastases, which may be related to the stimulating effect of androgens on the growth of lung cancer^12^. The 8th edition of the AJCC guidelines states that regardless of the patient's T or N stage, as long as distant metastases are present, their stage is stage IV. A study has shown that T and N stages cannot be ignored even in stage IV patients and that T and N stages indicate to a certain extent the malignancy of tumor cells and determine the treatment of NSCLC patients^13^. The study showed that the more advanced the T and N stages and the larger the tumor size, the worse the prognosis of the patients, probably because as the tumor size increases, the sensitivity of the tumor cells to treatments such as radiotherapy decreases^14^. Similarly, the higher the histological grade of the patient, the more aggressive the tumor tissue is, and accordingly the shorter the survival of the patients^15^. In patients with NSCLC, adenocarcinoma, compared to squamous carcinoma and other histological types, is more likely to develop liver metastases^16^. However, the histologic type is not one of the poor prognostic factors for NSCLC patients with liver metastases, which may be due to different chemotherapy regimens for patients with different histologic types. Among patients with single-site metastases from NSCLC, liver metastases have a relatively shorter median survival compared to brain or bone metastases. Patients with multisite metastases had a worse prognosis than single-site metastases, and among patients with multisite metastases, OS was reduced by 1 month or more in patients with liver metastases than in patients without liver metastases^17^.

The current standard treatment for NSCLC patients with liver metastases is systemic treatment, while for liver metastases, local treatments such as surgery, radiotherapy, radiofrequency ablation, and interventional therapy are available ^18^. Hepatectomy may be a treatment option when the patient’s liver metastasis is single lesions^19^. Sun et al. demonstrated that for NSCLC patients with only three metastases or less and single metastases, surgery can improve OS, as well as reduce the tumor burden of patients and reduce or eliminate tumor-induced complications, which can improve patient prognosis to some extent^20,21^. Although surgery significantly improves the prognosis, the vast majority of patients are lost to surgery at diagnosis, and only 3% of patients in our study underwent surgical treatment^22^. According to the previous opinion, patients with liver metastases from NSCLC have a relatively low response rate to chemotherapy, which may be since some patients with liver metastases have liver dysfunction and cannot tolerate chemotherapy^23^. A study by katsunori et al. showed a median survival of 6 months for patients receiving chemotherapy, which improved overall survival by 4 months compared to patients receiving only symptomatic supportive therapy^16^. Similarly, our study confirms that chemotherapy improves the prognosis of patients with liver metastases from NSCLC. Consistent with previous studies radiotherapy did not show an advantage in multivariate Cox regression analysis, suggesting that radiotherapy does not improve OS in patients with liver metastases from NSCLC, but radiotherapy can still be used as one of the palliative treatments to relieve pain, reduce complications and improve patients' quality of life^7^.

There are some limitations to our study. First, targeted therapies (TKIs) and immunotherapy are currently available for some populations of non-small cell lung cancer, but specific information on these treatments is not available in the SEER database, so we were unable to analyze whether these two treatments modalities could improve patient prognosis. Secondly, the study was a retrospective analysis, and details of the treatment modalities such as the surgical approach, chemotherapy regimen, and the site and dose of radiotherapy were not available. Third, the SEER database only collects data from the United States, and further research is needed to determine whether this nomogram is generalizable to other countries and ethnic groups.

## Conclusion

In brief, we established a prognostic nomogram for NSCLC patients with liver metastases by analyzing the basic information in the SEER database, which contains 11 variables including race, age, gender, grade, T stage, N stage, tumor size, bone metastases, brain metastases, surgery, and chemotherapy. Clinicians can use the prognostic nomogram to accurately assess the OS of patients, identify high-risk patients, and provide a reference for optimizing treatment plans.
